# Industry mobility and disability benefits in heavy manual jobs: A cohort study of Swedish construction workers

**DOI:** 10.5271/sjweh.3932

**Published:** 2021-03-31

**Authors:** Mia Söderberg, Mikael Stattin, Suzan JW Robroek, Alex Burdorf, Bengt Järvholm

**Affiliations:** Occupational and Environmental Medicine, School of Public Health and Community Medicine, Institute of Medicine, University of Gothenburg, Gothenburg, Sweden; Department of Sociology, Umeå University, Umeå, Sweden; Department of Public Health, Erasmus MC, University Medical Center, Rotterdam, The Netherlands; Department of Public Health and Clinical Medicine, Occupational Medicine, Umeå, Sweden

**Keywords:** construction industry, heavy work, Sweden, work ability

## Abstract

**Objectives:**

This study aimed to investigate whether change from the construction industry to work in other industries at age 45–55 years lowered risks of disability benefits (DB) later in life (60–64 years of age). We hypothesized that risks would be lowered the most among those changing from the heaviest occupations.

**Methods:**

The study included men employed in the construction industry during 1971–1993. We selected workers from the largest occupational groups in heavy (concrete workers and painters) and less heavy (drivers, electricians and foremen) occupations. The occurrence of DB in 1990–2015 was retrieved from national registers. Regression analyses were used to calculate relative risks (RR) of DB at 60–64 years, comparing those working in other industries to those still in the construction industry at the age of 45, 50 and 55 years.

**Results:**

Mobility away from the construction industry was related to lowered DB risks at 60–64 years in all selected occupations. Effects were most pronounced among those who, at 55 years of age, worked in an industry other than construction, with significantly reduced RR for DB among concrete workers [RR 0.63, 95% confidence interval (CI) 0.51–0.77], electricians (RR 0.61, 95% CI 0.47–0.77) and foremen (RR 0.78, 95% 0.63–0.96).

**Conclusions:**

Risks for DB at 60–64 years of age were reduced among those who changed from construction work to other industries. Notable reductions were observed among workers originating from both heavy and less heavy occupations, and future studies should explore other factors, in addition to heavy workload, as motivators for leaving the construction industry.

Longer life expectancy and the growing amount of elderly in proportion to active workers have created a need to expand work life duration (1). One way is to raise the statutory retirement age, but longer life expectancy does not necessarily equal delayed age-related disabilities (2). Above 60 years of age, most persons have some chronic disease. A study of Finnish municipality workers showed that 74% were diagnosed with a chronic disease around the age of 60 (3). Old age and chronic diseases are thus, unsurprisingly, the strongest predictors for premature labor market exit through disability benefits (DB) (4, 5).

As health and physical capacity decrease by age, older persons with heavy physical jobs should be more at risk of DB than others. The construction industry is a large occupational sector in many countries. In Sweden it employs about 6% of the workforce. The work is mostly physically demanding, including heavy physical workload, repetitive movements and working in demanding postures. Due to a high prevalence of musculoskeletal disorders, construction workers tend to leave the labor market earlier than others, often through DB programs (6–8).

Work modification – a process aimed at enhancing the match between job conditions and a worker’s resources – may promote a sustainable work life in those with reduced work ability. In persons with lower back pain (9) or injuries (10, 11), reduced work demands or tasks reassignments appear to be most effective. Work time control, eg, over breaks or flexible working hours, seem to benefit continued work at an older age in general (3). When such modifications are not possible in a current job setting, changing jobs may be the only option for improved job conditions and remaining at work. There is evidence that older persons in physically heavy occupations with musculoskeletal disorders, foremost lower back or spine disorders, change jobs more often than others (12–14). However, whether this expands working life is unknown, partly since such associations cannot be investigated by experimental studies, but has to rely on observational studies, which require a large study population and a long follow-up.

This study aimed to investigate whether changing from the construction industry to work in other industries, evaluated at the age of 45–55 years, lower the risks of DB at 60–64 years. We hypothesized that DB risks would be lower among those who changed to other industries, with the largest reductions occurring in those originating from the physically heaviest jobs.

## Methods

This study aimed to evaluate industry change and DB using the Swedish Construction Worker Cohort and Swedish national register data. The Construction Worker Cohort consists of 389 132 men and women, who were employed in the construction industry and attended health examinations during 1971–1993 through Bygghälsan (the Foundation for Occupational Safety and Health in the Swedish Construction Industry). The examinations were free of charge as part of the occupational health services, and all workers were invited on a routine basis at 2- to 5-year intervals. About 80% of those employed in the Swedish construction industry during that time have participated at least once. The cohort has been described in detail elsewhere (15).

DB are included in the Swedish sickness benefit welfare program and provide financial support for people with long-lasting reduced work ability. All residents aged 19–64 years, including unemployed persons, are covered. Eligibility requires ≥25% reduced work ability for at least one year as assessed by a physician. Benefits are either granted for a limited period of time with reassessments or, if the work ability is assessed as permanently reduced, benefits can be granted until entering old age retirement.

For the analyses, we selected the largest occupational groups with either heavy or lighter physical work. The job title used was determined by the title in the employment contract held at the time of the first medical examination. The division of heaviness was based on physical long-term cardiovascular load as a measure of work intensity, which was available through a job exposure matrix of heart rate measurements (16). The division also corresponds with practical knowledge of the construction industry. Two of the most common occupations – concrete workers and painters – were selected as the heaviest physical jobs based on cardiovascular load. Three of the other largest occupations were carpenters, electrician and foremen. We included electricians who have a lighter cardiovascular load than concrete workers and painters, even though they sometimes work in demanding postures. The foremen are mostly previous blue-collar workers, ie, they are from a similar socioeconomic group as the other three jobs, but with less manual tasks and therefore less load. We did not include carpenters as their work conditions are varied, and we wanted to include groups that could be generalized into heavy or lighter physical load. Instead, we included drivers of trucks, cranes and heavy equipment. Electricians and foremen have less load but typically also higher qualifications. By including drivers, we could observe a group with a similar level of qualifications to concrete workers and painters but who work in sitting postures and on average have a low cardiovascular load.

We compared the occurrence of DB at age 60–64 years among men in the selected occupations, who worked in another industry the year they turned 45, 50 and 55 years of age, to those who remained in the construction industry. Change of industry could have occurred at any time between inclusion and the evaluated ages, but since many, especially in younger ages, change back and forth between industries, we defined “change of industry” as not working in construction the calendar year that the individual turned 45, 50 or 55 years of age. Hence, change of industry was defined as yes/no at the evaluated age, ie, persons who left construction at age 45 could be counted as being in the industry at age 55 years if they returned to construction. Information on type of industry after inclusion and timing of granted DB between 1990–2015 was available through the national register the longitudinal integrated database for health insurance and labor market studies (LISA), provided by Statistics Sweden, which covers all Swedish residents aged ≥16 years. The LISA register was established in 1990. Due to the Sweden’s unique personal identification number, the register data could be matched to all studied men on an individual level.

Comparing occurrence of DB between those who had changed industry with those who remained in the construction industry, evaluated at the ages of 45, 50 and 55 years, meant that three sub-cohorts were used (table 1). Each sub-cohort included all uncensored persons from inclusion at clinical examination until age of evaluated industry change (1990–2010). The ages 45, 50 and 55 were chosen since physical capacity and health starts to decline at these ages. The follow-up started in the calendar age when the men were 60 years, if they lived in Sweden, currently did not uphold DB and were registered in LISA as having a job (full or part-time). Each individual was followed until: (i) the first occurrence of DB, (ii) the calendar year they turned 65 years, (iii) death, (iv) emigration, or (v) 31 December 2015. For example, a person who was 45 years in 1991 was followed until 2006–2010 at the latest. The analyses were restricted to men as there were too few women in the selected occupations to make analyses of women feasible. The age window 60–64 years constitutes an age span in close proximity to statutory retirement age where chronic diseases and DB are most common.

**Table 1:** Number of men who were included at follow-up and received disability benefits (DB) at age 60–64 years, by job at first health control and by industry at 45, 50 and 55 years of age.

Job at inclusion	In construction industry	Industry evaluated at age 45		Industry evaluated at age 50		Industry evaluated at age 55	
		
		DB cases per men at risk at follow-up	Sector mobility (%)	DB cases per men at risk at follow-up	Sector mobility (%)	DB cases per men at risk at follow-up	Sector mobility (%)
Concrete worker	Yes	142/2345		497/3516		922/3881	
	No	92/1753	42.8	237/2630	42.8	403/3340	46.3
Painter	Yes	134/2948		348/2957		522/4453	
	No	53/1359	31.6	152/2660	47.4	218/2136	32.4
Drivers	Yes	75/1901		260/3892		462/3683	
	No	52/1720	47.5	205/1829	32.0	354/3402	48.0
Electrician	Yes	135/4723		360/5779		567/6516	
	No	99/3908	45.3	220/4839	45.6	300/5385	45.2
Foremen	Yes	99/3704		320/5581		305/6686	
	No	62/2516	40.5	192/4039	42.0	543/5199	43.7
Number of men at 60 years of age	Yes	585/15 622		1785/21 725		2778/25 219	
	No	358/11 256	41.9	1006/15 997	42.4	1818/19 462	43.6

Relative risks (RR) and 95% confidence intervals (95% CI) for DB between those who changed from the construction industry and those who remained were estimated by a negative binomial regression model of incidence rates using log link. Persons who stayed in the construction industry were the reference. The analyses were adjusted for age between 60–64 years (1-year intervals), smoking habits at health examination [non-, ex-, moderate, and heavy smokers (15+ cigarettes)] and body mass index (BMI) (18.5–24.9, 25–29.9, and 30–34.9 kg/m^2^). Persons with unknown smoking habits (6.7%) and unknown BMI or BMI <18.5 or ≥35 kg/m^2^ were excluded (3.1%). A 95% CI not including 1 was considered as statistically significant. The Ethical Review Board at Umeå University approved this study (2016/308-31).

We carried out sensitivity analyses by expanding change of industry to the age intervals 44–46, 49-51 and 54–56 years of age, but these analyses displayed similar results as those presented and are not included in this paper. To study a possible influence of poor health on risks of DB at 60–64 years of age, additional analyses were restricted to persons that had not been hospitalized around the evaluated ages of change in industry, eg, not been hospitalized between 44–46 years for those observed at 45 years of age. The results were similar as in the main analyses and are not presented.

## Results

Table 1 shows the number of DB cases by sector mobility, stratified by age cohorts. The proportion of workers who, at the age of evaluation, worked in another industry was lowest among painters, while the other job categories displayed similar proportions of industry mobility (43–50%). Characteristics of the sub-cohorts at the first clinical examination and at follow-up are available in the supplementary material (www.sjweh.fi/show_abstract.php?abstract_id=3932), appendix 1a-c.

**Figure 1:**
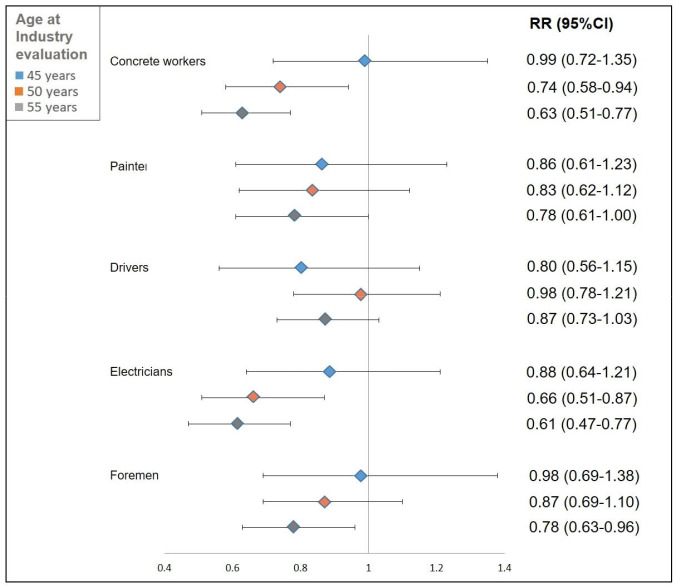
Relative risk of disability benefits between 60–64 years of age among men, depending on job at first health control and by change to other industries at 45, 50 and 55 years of age.

In figure 1, RR are displayed for different age groups. Although several results were non-significant, all analyses displayed reduced RR for DB at 60–64 years of age among those who had shifted out of the construction industry compared to those who had remained.

Results also revealed differences between the selected occupations and ages at evaluated industry change. Among concrete workers, those who worked in another industry had statistically significantly lowered DB risks at 60–64 years in analyses that examined a move from the construction industry at age 50 (RR 0.74, 95% CI 0.58–0.94) and 55 (RR 0.63, 95% CI 0.51–0.77), compared to those who remained in the industry. Similarly, results for electricians showed lowered estimates for workers who changed industry at age 50 (RR 0.66, 95% CI 0.51–0.87) and 55 (RR 0.61, 95% CI 0.47–0.77). Despite overall lower DB risks among foremen transferring to other industries, only results of industry change evaluated at 55 years were statistically significant (RR 0.78, 95% 0.63–0.96).

For persons that worked in another industry at age 45, all risk estimates were <1 but non-significant and CI were wide. Industry change evaluation at age 50 displayed lowered DB risks than those who remained in construction, which was further lowered at aged 55 compared to 50 years. The age effects of lower DB risks in older ages were observed in all occupations except drivers and appeared most conspicuous in concrete workers, electricians and foremen.

## Discussion

This study showed that movement out of the construction industry to other industries was related to lowered risks for DB at 60–64 years of age. Results were most pronounced among those whose industry change was evaluated at age 55, with considerably lowered RR among concrete workers, electricians and foremen.

We investigated the study objectives in five occupational groups of varied physical workload heaviness. Industry change was related to lowered DB risks in all groups, a finding which concurs with similar studies (12–14), as mobility away from heavy occupations presumably reduces work load, improves health, or at least enhances the match between job demands and a worker’s capacity. The reference group, who remained in heavy jobs, face higher risks for onset of or worsened chronic diseases.

In contrast to our hypothesis, that change from the heaviest jobs would be the most beneficial: the largest DB risk reductions were found in both heavy (concrete workers) and less heavy occupations (electricians and foremen). In a study based on the same cohort, concrete workers and painters represented occupations with the most lost working years due to DB, while foremen and electricians displayed the least lost working years (8). Thus, it seemed probable the largest DB reduction would occur among concrete workers and painters. Other similar studies have not analyzed occupations according to variations of workload (12, 13), and it is unknown if DB risks generally are the most lowered in those leaving the heavier occupations.

Results among the heaviest occupations also displayed deviations as significant lowered risks of DB among concrete workers, but rather small and non-significant effects among painters, were observed. A suggested explanation is that painters have better opportunities for self-employment, through which they remain in the same industry but with better control possibilities over job offers, work hours and breaks, which enhance sustainable work ability (3). Painters had the lowest percentage of industry mobility, providing some support to this theory.

Among the lighter occupations, electricians displayed the most lowered DB risks. Among foremen, industry change was also related to statistically lowered risks of DB, but effects were smaller and only statistically significant in those evaluated at age 55. We lack information on reasons for industry change and can only speculate. Electricians and foremen typically not only have lighter work tasks but also higher qualifications. If leaving the construction industry, less straining jobs may be available, eg, electricians may move to maintenance and foremen become instructors. Industry mobility may then, at least partly, be determined by pull factors to attractive jobs rather than poor health and heavy work. Among drivers, an occupation with lighter work tasks but lower qualification requirements, DB risk were small and non-significant in all age groups. The different patterns between the selected occupations indicate that several factors other than heavy workload, are important. Since DB is likely determined by conditions in the new job, we examined which industries the workers changed to (supplementary appendix 2a-c). However, since we only had access to industry, not occupation, there is little precision on new work conditions. Most notably, there was high industry mobility to the financial sector among foremen (around 43%), likely since they have higher qualifications than other occupations. Otherwise, patterns were similar for most occupational groups and added little explanation for differences in DB risks.

The results displayed age effects, as those whose industry mobility were evaluated at 45 years of age generally had the least reduced DB risks, while those working outside the construction industry at age 55 displayed the most reduced risks. Industry change was defined as not working in the construction industry the calendar year the worker turned 45, 50 or 55 years of age. It could be, foremost in younger ages, that workers change back and forth and the industry change is not permanent. We could only study industry change from 1990 when the LISA register started. Some men changed industry a few times during the study period. Therefore, we also studied men who had the same job during 3-year periods around 45, 50 and 55 years, but the findings were similar.

Most occupations displayed high numbers of workers changing industry, even among those evaluated at age 45. In some, industry change may relate to the period (ie, the 1970s and 1980s), when it was quite common for construction workers to be employed in short-term contracts that ended when the building project ended. If no other construction work was available, the worker would seek employment in other industries requiring similar skill levels. Repeated changes, foremost in older ages, may be a sign of trying other jobs due to health problems, but also a sign of skills and health as such workers have more possibilities to find other jobs with better pay. If health is a decisive factor, the time between change and follow-up is of importance. Those evaluated at age 55 had the most reduced risks and were in closest proximity to follow-up, which started at 60 years of age. As health starts to decline sharply around age 55 in physically demanding jobs (12), industry change in this age group would be most driven by health and thus benefit the most. Contrary, if good health is positively correlated to industry change, it will also have a larger influence for reduced DB risk in this age group, as poor health is less common among younger workers. Driver was the only occupation in which no age effects appeared, perhaps because the work conditions are lighter but advancement outside the construction industry is limited.

Results may also relate to selection effects. Workers who succeeded in changing jobs may represent a selection of individuals with better health and a personality type, who want a long working life and choose to invest in a more fulfilling and stimulating job. After changing industry, additional positive effects may have followed and reignited the motivation to remain at work. The presence of such persons will over-estimate positive effects of changing industry. In contrast, if changing industry due to chronic diseases, the effect could be underestimated due to higher prevalence of poor health. The presented results are similar to those in the sensitivity analyses, in which all hospitalized persons were excluded, and poor health may have less effect on underestimations. However, most disorders among construction workers are treated in outpatient care. There are no national registers of outpatient care that cover our observation period.

Our observation period stretches over a large time span, during which incidence of DB has varied greatly in this cohort. Welfare legislations have also varied over this time, and – during some periods – eligibility criteria for DB, reassessment of work ability, and return to work among persons >60 years have either been more generous or stricter (17). Since our follow-up is determined by age and not calendar year, it is difficult to determine the effects of welfare legislations. Furthermore, during times of recession, it is common that workers with lower capacity and chronic diseases are pushed out of their jobs, underestimating the effects of industry mobility (14). But in periods of recession, short-term sick leave tends to decrease due to fear of losing one’s job (18). As a consequence, long-term sick-leave and DB increase, as many with poor health continue working until onset of chronic illnesses. Meanwhile, in times of economic boom and more work opportunities, many may change industry for reasons unrelated to health. Variations in societal economics can therefore lead to both under- and overestimation of DB risk among those changing industry.

### Limitations

The cohort consisted of male blue-collar workers in the construction sector, and results may not be generalized to women or other occupational sectors. This is a major limitation since there is evidence that construction workers have a higher risk for DB, even compared to other blue-collar workers in eg, metal and chemical industries, or mining (19). We were also unable to examine risk reduction of DB from mobility between employers while remaining in the same industry or if industry change resulted in changed job conditions. Another limitation is that we did not assess if persons granted DB at 60–64 years returned to work. Given the heaviness of construction work, it is unlikely that someone >60 years of age would resume work if entering DB programs, but it cannot be ruled out.

### Strengths

This study examined the effects of moving out of the construction industry using a large cohort with a long follow-up time and individual information on several important covariates.

The cohort size allowed division into sub-cohorts by occupational groups with variations of physical workload. Since age is an important covariate in this context, we also took this into consideration by division into age-specified cohorts. Due to Sweden’s usage of unique personality numbers and high-quality national registers, we also have accurate data on timing of DB and industry. Finally, considering that low socioeconomic status and less education consistently has been identified as a strong determinant for DB and lost working years (20), it was also beneficial to have access to a large cohort of only construction industry workers.

### Concluding remarks

Our results found reduced DB risks at 60–64 years of age among those who changed from construction work to other industries. Notable reductions were observed in both heavy and less heavy jobs, indicating that factors other than physically demanding work could be important for industry mobility and DB; this needs to be explored further. Still, supportive functions that facilitate job mobility close to statutory retirement age may increase work participation in older workers.

## Supplementary material

Supplementary material
