# Supplementary material for: Industry mobility and disability benefits in heavy manual jobs: A cohort study of Swedish construction workers
Source: Scand J Work Environ Health. 2021 Mar 31;47(3):217–23. doi: 10.5271/sjweh.3932 (PMC8126441; doi:10.5271/sjweh.3932)
Supplement: Supplementary material [file SJWEH-47-217-S001.pdf]

Industry mobility and disability benefits in heavy manual jobs: A cohort study of Swedish construction workers [1]

by Mia Söderberg, PhD,[2] Mikael Stattin, PhD, Suzan JW Robroek, PhD, Alex Burdorf, PhD, Bengt Järvholm, PhD

1. *Supplementary material*
2. *Correspondence to: Mia Söderberg, Occupational and Environmental Medicine, School of Public Health and Community Medicine, Institute of Medicine, University of Gothenburg, Sweden. [E-mail: mia.soderberg@amm.gu.se]*

Appendix 1a. *Characteristics of the sub-cohort by 45 years of age at evaluated industry, at the 1st examination and at follow-up*

	**At first clinical examination**								**At follow-up at 60-64 years of age**	
**Age cohort**	Occupation at first health examination	In construction industry	N (%)	Average age at first health examination	BMI >25 -29.9 kg/m^2 N (%)	BMI 30-34.9 kg/m^2 N (%)	Non-Smoker N (%)	Heavy-Smoker N (%)	Uncensored at 60 years of age, N (%)	Age when granted DP during follow-up, mean (sd)
**45 years**	*Concrete worker*	Yes	5319 (52.5)	25.5 (5.7)	1363 (25.6)	160 (3.0)	2390 (44.9)	920 (17.3)	2974 (55.9)	61.6 (1.3)
		No	4816 (47.5)	24.7 (5.4)	1205 (25.0)	175 (3.6)	2206 (45.8)	932 (19.4)	3063 (63.6)	61.5 (1.3)
	Painter	Yes	6869 (64.6)	24.2 (4.9)	1199 (17.5)	118 (1.7)	3456 (50.3)	891 (13.0)	3921 (57.1)	61.6 (1.4)
		No	3767 (35.4)	23.6 (4.8)	712 (18.9)	93 (2.5)	1920 (51.0)	596 (15.8)	2408 (63.9)	62.0 (1.6)
	Drivers	Yes	3150 (50.3)	28.8 (6.0)	1060 (33.7)	185 (5.9)	1199 (38.1)	637 (20.2)	1249 (39.7)	61.8 (1.5)
		No	3114 (49.7)	27.5 (5.6)	979 (31.4)	185 (5.9)	1208 (38.8)	665 (21.4)	1394 (44.8)	61.9 (1.4)
	Electrician	Yes	11301 (53.0)	24.6 (4.9)	2104 (18.6)	201 (1.8)	7064 (62.5)	1028 (9.1)	6577 (58.2)	61.7 (1.5)
		No	10021 (47.0)	24.2 (4.6)	1872 (18.7)	167 (1.7)	6387 (63.7)	890 (8.9)	6113 (61.0)	61.9 (1.8)
	Foremen	Yes	5633 (55.1)	27.8 (5.4)	1287 (22.9)	100 (1.8)	3087 (54.6)	721 (12.8)	1929 (34.2)	62.0 (1.9)
		No	4595 (44.9)	26.8 (5.1)	944 (20.5)	81 (1.8)	2747 (59.8)	498 (10.8)	2079 (45.2)	61.8 (1.6)

Appendix 1b. *Characteristics of the sub-cohort by 50 years of age at evaluated industry, at the 1st examination and at follow-up*

	**At first clinical examination**								**At follow-up at 60-64 years of age**	
**Age cohort**	Occupation at first health examination	In construction industry	N (%)	Average age at first health examination	BMI >25-29.9 kg/m^2 N (%)	BMI 30-34.9 kg/m^2 N (%)	Non-Smoker N (%)	Heavy-Smoker N (%)	Uncensored at 60 years of age, N (%)	Age when granted DP, mean (sd)
**50 years**	*Concrete worker*	Yes	5939 (54.6)	27.9 (6.2)	1668 (28.1)	177 (3.0)	2220 (37.4)	1085 (18.3)	2423 (40.8)	62.0 (1.5)
		No	4939 (45.4)	27.1 (6.4)	1373 (27.8)	184 (3.7)	1891 (38.3)	958 (19.4)	2309 (48.8)	61.8 (1.5)
	Painter	Yes	6659 (65.4)	26.2 (5.8)	1267 (19.0)	131 (2.0)	2963 (44.5)	896 (13.5)	2767 (41.6)	62.0 (1.7)
		No	3525 (34.6)	25.7 (5.8)	735 (20.9)	81 (2.3)	1513 (42.9)	557 (15.8)	1696 (48.1)	61.9 (1.7)
	Drivers	Yes	4097 (51.5)	31.3 (6.6)	1527 (37.3)	266 (6.5)	1385 (33.8)	769 (18.8)	1140 (27.8)	62.1 (1.5)
		No	3861 (48.5)	29.6 (6.2)	1288 (33.4)	230 (6.0)	1301 (33.7)	837 (21.7)	1201 (31.1)	62.1 (1.7)
	Electrician	Yes	10086 (53.3)	26.5 (5.7)	2094 (20.8)	193 (1.9)	5414 (53.7)	1118 (11.1)	4307 (42.7)	62.0 (1.8)
		No	8821 (46.7)	26.0 (5.5)	1800 (20.3)	168 (1.9)	4910 (55.5)	1795 (10.4)	3982 (45.0)	62.0 (1.6)
	Foremen	Yes	7184 (55.4)	30.2 (6.1)	1885 (26.2)	178 (2.5)	3445 (48.0)	1057 (14.7)	1603 (22.3)	62.2 (2.0)
		No	5789 (44.6)	28.9 (5.9)	1361 (23.5)	130 (2.3)	2966 (51.2)	794 (13.7)	1750 (30.2)	62.0 (2.0)

Appendix 1c. *Characteristics of the sub-cohort by 55 years of age at evaluated industry, at the 1st examination and at follow-up*

	At first clinical examination								At follow-up at 60-64 years of age	
**Age cohort**	Occupation at first health examination	In construction industry	N (%)	Average age at first health examination	BMI >25-29.9 kg/m^2 N (%)	BMI 30-34.9 kg/m^2 N (%)	Non-Smoker N (%)	Heavy-Smoker N (%)	Uncensored at 60 years of age, N (%)	Age when granted DP, mean (sd)
**55 years**	*Concrete worker*	Yes	5712 (56.7)	31.0 (6.8)	1780 (31.2)	187 (3.2)	1816 (31.2)	932 (16.3)	1831 (19.8)	61.8 (1.4)
		No	4356 (43.3)	29.9 (7.0)	1279 (29.4)	165 (3.8)	1380 (31.7)	815 (18.7)	1016 (23.3)	61.9 (1.1)
	Painter	Yes	5776 (66.8)	28.4 (6.7)	1161 (20.1)	126 (2.2)	2298 (39.8)	757 (12.6)	1323 (22.9)	61.9 (1.6)
		No	2872 (33.2)	27.9 (6.5)	642 (22.4)	61 (2.1)	1102 (38.4)	421 (14.7)	736 (25.6)	62.0 (1.8)
	Drivers	Yes	4337 (51.3)	33.3 (7.2)	1676 (38.6)	264 (6.1)	1354 (31.2)	757 (17.5)	654 (15.1)	61.9 (1.0)
		No	4113 (48.7)	31.7 (7.0)	1488 (36.2)	274 (6.7)	1280 (31.1)	874 (20.6)	711 (17.3)	62.1 (1.8)
	Electrician	Yes	8406 (53.9)	28.6 (6.6)	1854 (22.1)	170 (2.0)	3903 (46.4)	976 (11.6)	1890 (22.5)	62.0 (1.7)
		No	7172 (46.0)	27.7 (6.4)	1568 (21.9)	146 (2.0)	3505 (48.9)	789 (11.0)	1787 (24.9)	62.0 (1.6)
	Foremen	Yes	7505 (55.1)	32.4 (6.8)	2190 (25.5)	210 (2.8)	3275 (43.6)	1118 (14.9)	819 (10.9)	62.0 (1.9)
		No	6126 (44.9)	30.8 (6.6)	1563 (25.5)	167 (2.7)	2798 (45.7)	930 (15.2)	927 (15.1)	62.0 (1.9)

Appendix 2a. New industries for worker evaluated at 45 years of age

Occupation at first clinical examination	Concrete workers		Painters		Drivers		Electricians		Foremen	
	n	%	n	%	n	%	n	%	n	%
New industry										
− Agriculture, forestry, fishery	182	3.78	83	2.20	239	7.68	138	1.38	71	1.55
− Manufacturing	1358	28.20	794	21.08	733	23.54	2560	25.55	826	17.98
− Energy production/Water supply/waste disposal	151	3.14	44	1.17	108	3.47	1139	11.37	141	3.07
− Trade/communication	1133	23.53	795	21.10	1222	39.24	2261	22.56	790	17.19
− Financial work	961	19.95	1067	28.32	475	15.25	2394	23.89	1992	43.35
− Education / Research	246	5.11	251	6.66	80	2.57	465	4.64	145	3.16
− Health and social work	242	5.02	254	6.74	72	2.31	286	2.85	116	2.52
− Culture work	317	6.58	296	7.86	94	3.02	405	4.04	126	2.74
− Administration	226	4.69	183	4.86	91	2.92	373	3.72	388	8.44
Sum of workers who changed industries	4816		3767		3114		10 021		4595	

Appendix 2b. New industries for worker evaluated at 50 years of age

Occupation at first clinical examination	Concrete workers		Painters		Drivers		Electricians		Foremen	
	n	%	n	%	n	%	n	%	n	%
New industry										
− Agriculture, forestry, fishery	221	4.47	89	2.52	270	6.99	131	1.48	115	1.99
− Manufacturing	1334	27.01	657	18.64	888	23.00	2118	23.93	950	16.41
− Energy production/Water supply/waste disposal	148	3.00	36	1.02	154	3.99	1132	12.79	192	3.32
− Trade/communication	1068	21.62	662	18.78	1450	37.56	1835	20.73	954	16.48
− Financial work	1035	20.96	1097	31.12	608	15.75	2125	24.01	2537	43.82
− Education / Research	276	5.59	264	7.49	120	3.11	499	5.64	166	2.87
− Health and social work	244	4.94	267	7.57	102	2.64	291	3.29	139	2.40
− Culture work	381	7.71	275	7.80	145	3.76	372	4.20	185	3.20
− Administration	232	4.70	178	5.05	124	3.21	348	3.93	551	9.52
Sum of workers who changed industries	4939		3525		3861		8851		5789	

Appendix 2c. New industries for worker evaluated at 55 years of age

Occupation at first clinical examination	Concrete workers		Painters		Drivers		Electricians		Foremen	
	n	%	n	%	n	%	n	%	n	%
New industry										
− Agriculture, forestry, fishery	191	4.38	84	2.92	254	6.18	122	1.70	114	1.86
− Manufacturing	1131	25.96	501	17.44	947	23.02	1666	23.23	974	15.90
− Energy production/Water supply/waste disposal	135	3.10	24	0.84	174	4.23	1028	14.33	230	3.75
− Trade/communication	858	19.70	492	17.13	1461	35.52	1308	18.24	943	15.39
− Financial work	977	22.43	941	32.76	719	17.48	1784	24.87	2645	43.18
− Education / Research	235	5.39	216	7.52	135	3.28	414	5.77	168	2.74
− Health and social work	191	4.38	220	7.66	94	2.29	241	3.36	159	2.60
− Culture work	388	8.91	235	8.18	199	4.84	327	4.56	239	3.90
− Administration	250	5.74	159	5.54	130	3.16	282	3.93	654	10.68
Sum of workers who changed industries	4356		2872		4113		7172		6126	
